# Crystal structure of 2-chloro-1-(3-ethyl-2,6-di­phenyl­piperidin-1-yl)ethanone

**DOI:** 10.1107/S2056989015000444

**Published:** 2015-01-21

**Authors:** V. Shreevidhyaa Suressh, B. K. Revathi, S. Abdul Basheer, S. Ponnuswamy, G. Usha

**Affiliations:** a Department of Physics, Anna Adarsh College for Women, Chennai-40, Tamilnadu, India; b PG and Research Department of Physics, Queen Mary’s College, Chennai-4, Tamilnadu, India; c PG and Research Department of Chemistry, Government Arts College, Coimbatore 18, Tamilnadu, India

**Keywords:** crystal structure, 2-chloro-1-(3-ethyl-2,6-di­phenyl­piperidin-1-yl)ethanone, biological activity, piperidine derivative

## Abstract

In the racemic title compound, C_21_H_24_ClNO, the dihedral angle between the planes of the benzene rings is 86.52 (14)° and those between the benzene rings and the piperidine ring are 61.66 (14) and 86.39 (14)°. The piperidine ring adopts a twisted boat conformation. No directional inter­actions could be detected in the crystal.

## Related literature   

For the biological activity of piperidine derivatives, see: Nalanishi *et al.* (1974 ▸); Robinson (1973 ▸); Mobio *et al.* (1989 ▸); Parthiban *et al.* (2009 ▸).
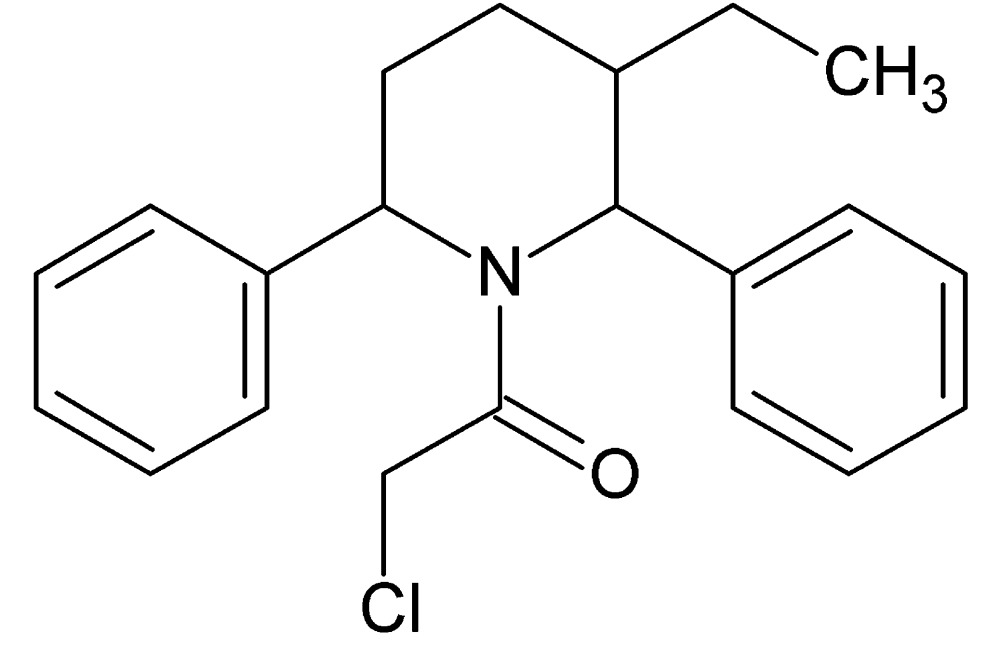


## Experimental   

### Crystal data  

C_21_H_24_ClNO
*M* *_r_* = 341.86
Monoclinic, 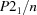

*a* = 8.5971 (9) Å
*b* = 12.9080 (13) Å
*c* = 17.1114 (16) Å
β = 100.501 (5)°
*V* = 1867.1 (3) Å^3^
*Z* = 4
Mo *K*α radiation
μ = 0.21 mm^−1^
*T* = 293 K
0.25 × 0.23 × 0.23 mm

### Data collection  

Bruker Kappa APEXII CCD diffractometer
Absorption correction: multi-scan (*SADABS*; Bruker, 2008 ▸) *T*_min_ = 0.949, *T*_max_ = 0.953
17549 measured reflections
4639 independent reflections
2930 reflections with *I* > 2σ(*I*)
*R*_int_ = 0.027

### Refinement  

*R*[*F* ^2^ > 2σ(*F* ^2^)] = 0.064
*wR*(*F* ^2^) = 0.226
*S* = 1.01
4639 reflections
217 parameters
H-atom parameters constrained
Δρ_max_ = 0.61 e Å^−3^
Δρ_min_ = −0.43 e Å^−3^

### 

Data collection: *APEX2* (Bruker, 2008 ▸); cell refinement: *SAINT* (Bruker, 2008 ▸); data reduction: *SAINT*; program(s) used to solve structure: *SHELXS97* (Sheldrick, 2008 ▸); program(s) used to refine structure: *SHELXL97* (Sheldrick, 2008 ▸, 2015 ▸); molecular graphics: *ORTEP-3 for Windows* (Farrugia, 2012 ▸); software used to prepare material for publication: *SHELXL97* and *PLATON* (Spek, 2009 ▸).

## Supplementary Material

Crystal structure: contains datablock(s) I, New_Global_Publ_Block. DOI: 10.1107/S2056989015000444/zs2323sup1.cif

Structure factors: contains datablock(s) I. DOI: 10.1107/S2056989015000444/zs2323Isup2.hkl

Supporting information file. DOI: 10.1107/S2056989015000444/zs2323Isup3.cml

**Fig. 1.**
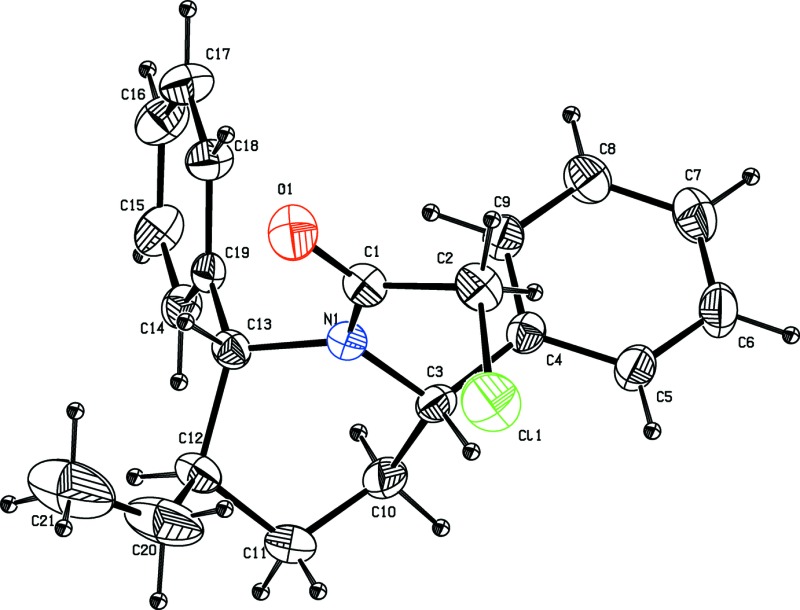
The molecular structure of the title compound showing atom numbering, with displacement ellipsoids drawn at the 30% probability level.

. DOI: 10.1107/S2056989015000444/zs2323fig1.tif
The mol­ecular structure of the title compound showing atom numbering, with displacement ellipsoids drawn at the 30% probability level.

**Fig. 2.**
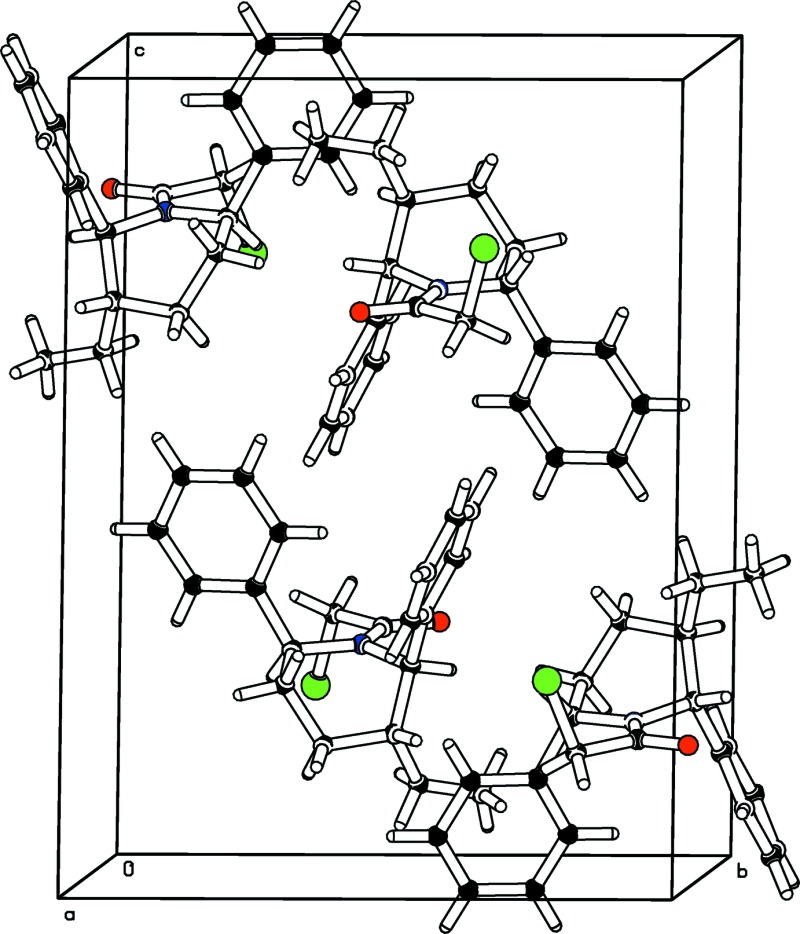
The packing of the molecules in the unit cell.

. DOI: 10.1107/S2056989015000444/zs2323fig2.tif
The packing of the mol­ecules in the unit cell.

CCDC reference: 1042840

Additional supporting information:  crystallographic information; 3D view; checkCIF report

## supplementary crystallographic information

### S1. Comment

Piperidine derivatives have immense biological significance and show blood cholesterol lowering activity (Nalanishi *et al.*, 1974; Parthiban *et al.*, 2009). The piperidine moiety is the basic unit commonly found in natural alkaloids. These compounds are observed to exhibit antihistamine, anaesthetic and tranquilizer activities (Robinson, 1973). They also exhibit antifungal and antibacterial activities (Mobio *et al.*, 1989).

In the stucture of the racemic title compound, C_21_H_24_ClNO (Fig. 1) the C—C bond lengths and bond angles are in the normal range and comparable with the literature values. The sum of the bond angles around the nitrogen atom N1 is 359.54 (1)° which indicates sp^2^ hybridization. The benzene ring C4/C5/C6/C7/C8/C9 is bisectionally [59.92 (2)°] attached to the piperidine ring and the benzene ring C14/C15/C16/C17/C18/C19 is axiallly [9.27 (2)°] attached to the piperidine ring. The dihedral angle between the benzene rings is 86.52 (14)° and those between the benzene rings and the piperidine ring are 61.66 (14) and 86.39 (14)°, respectively. The symmetric bond angles [C11—C12—C20 = 107.4 (3) Å and C20—C12—C13 = 110.3 (2) Å] indicate that the ethyl group is coplanar with the piperidine ring. The piperidine ring adopts a twisted boat conformation with puckering parameters of q2 = 0.691 (3) Å, θ2 = 94.4 (2)°, q3 = -0.053 (3) Å, QT = 0.693 (3) Å and smallest asymmetry parameter D2(C3) (Nardelli, 1983) is 0.1078 (1) Å. In the crystal there are no formal hydrogen bonds or inter-ring π–π interactions present (Fig. 2).

### S2. Experimental

A mixture of t-3-methyl-r-2,c-6-diphenylpiperidine (5 mmol), chloroacetyl chloride (20 mmol) and triethylamine (20 mmol) in anhydrous benzene (20 ml) was stirred at room temperature. The precipitated ammonium salt was filtered and the resulting solution was washed with water and bicarbonate solution (4x10 ml). Finally, the benzene solution was dried over anhydrous sodium sulphate and concentrated. The pasty mass was purified by crystallization from a mixture of petroleum ether (60–80 °C) and ethyl acetate in the ratio of 95:5.

### S3. Refinement

Hydrogen atoms were positioned geometrically and treated as riding on their parent atoms, with C—H distances of 0.93–0.98 Å, with *U*_iso_(H)= 1.5*U*_eq_(C-methyl) and *U*_iso_(H) = 1.2*U*_eq_(C) for other H atoms.

### Crystal data

**Table:**

C_21_H_24_ClNO	*F*(000) = 728
*M**_r_* = 341.86	*D*_x_ = 1.216 Mg m^−^^3^
Monoclinic, *P*2_1_/*n*	Mo *K*α radiation, λ = 0.71073 Å
Hall symbol: -P 2yn	Cell parameters from 4639 reflections
*a* = 8.5971 (9) Å	θ = 2.0–28.3°
*b* = 12.9080 (13) Å	µ = 0.21 mm^−^^1^
*c* = 17.1114 (16) Å	*T* = 293 K
β = 100.501 (5)°	Block, colourless
*V* = 1867.1 (3) Å^3^	0.25 × 0.23 × 0.23 mm
*Z* = 4	

### Data collection

**Table:**

Bruker Kappa APEXII CCD diffractometer	4639 independent reflections
Radiation source: fine-focus sealed tube	2930 reflections with *I* > 2σ(*I*)
Graphite monochromator	*R*_int_ = 0.027
ω and φ scan	θ_max_ = 28.3°, θ_min_ = 2.0°
Absorption correction: multi-scan (*SADABS*; Bruker, 2008)	*h* = −10→11
*T*_min_ = 0.949, *T*_max_ = 0.953	*k* = −17→16
17549 measured reflections	*l* = −22→22

### Refinement

**Table:**

Refinement on *F*^2^	Primary atom site location: structure-invariant direct methods
Least-squares matrix: full	Secondary atom site location: difference Fourier map
*R*[*F*^2^ > 2σ(*F*^2^)] = 0.064	Hydrogen site location: inferred from neighbouring sites
*wR*(*F*^2^) = 0.226	H-atom parameters constrained
*S* = 1.01	*w* = 1/[σ^2^(*F*_o_^2^) + (0.1098*P*)^2^ + 0.7138*P*] where *P* = (*F*_o_^2^ + 2*F*_c_^2^)/3
4639 reflections	(Δ/σ)_max_ < 0.001
217 parameters	Δρ_max_ = 0.61 e Å^−^^3^
0 restraints	Δρ_min_ = −0.43 e Å^−^^3^

### Special details

**Table:**

Geometry. All e.s.d.'s (except the e.s.d. in the dihedral angle between two l.s. planes) are estimated using the full covariance matrix. The cell e.s.d.'s are taken into account individually in the estimation of e.s.d.'s in distances, angles and torsion angles; correlations between e.s.d.'s in cell parameters are only used when they are defined by crystal symmetry. An approximate (isotropic) treatment of cell e.s.d.'s is used for estimating e.s.d.'s involving l.s. planes.
Refinement. Refinement of *F*^2^ against ALL reflections. The weighted *R*-factor *wR* and goodness of fit *S* are based on *F*^2^, conventional *R*-factors *R* are based on *F*, with *F* set to zero for negative *F*^2^. The threshold expression of *F*^2^ > σ(*F*^2^) is used only for calculating *R*-factors(gt) *etc*. and is not relevant to the choice of reflections for refinement. *R*-factors based on *F*^2^ are statistically about twice as large as those based on *F*, and *R*- factors based on ALL data will be even larger.

### Fractional atomic coordinates and isotropic or equivalent isotropic displacement parameters (Å^2^)

**Table:**

	*x*	*y*	*z*	*U*_iso_*/*U*_eq_	
C1	0.3154 (3)	0.4581 (2)	0.29469 (14)	0.0637 (6)	
C2	0.2091 (3)	0.3683 (2)	0.30518 (16)	0.0740 (7)	
H2A	0.1359	0.3886	0.3394	0.089*	
H2B	0.2721	0.3109	0.3301	0.089*	
C3	0.5246 (3)	0.33072 (17)	0.27780 (13)	0.0581 (5)	
H3	0.4458	0.2917	0.2406	0.070*	
C4	0.5527 (2)	0.27291 (17)	0.35598 (14)	0.0564 (5)	
C5	0.5133 (3)	0.1687 (2)	0.35639 (18)	0.0752 (7)	
H5	0.4631	0.1372	0.3097	0.090*	
C6	0.5478 (4)	0.1116 (2)	0.4253 (2)	0.0952 (10)	
H6	0.5224	0.0415	0.4246	0.114*	
C7	0.6198 (4)	0.1576 (3)	0.4952 (2)	0.0900 (9)	
H7	0.6427	0.1188	0.5417	0.108*	
C8	0.6573 (3)	0.2605 (2)	0.49595 (17)	0.0792 (7)	
H8	0.7048	0.2919	0.5432	0.095*	
C9	0.6250 (3)	0.3181 (2)	0.42674 (15)	0.0674 (6)	
H9	0.6521	0.3879	0.4277	0.081*	
C10	0.6770 (3)	0.3332 (2)	0.24373 (17)	0.0767 (8)	
H10A	0.7614	0.3630	0.2826	0.092*	
H10B	0.7072	0.2630	0.2327	0.092*	
C11	0.6550 (5)	0.3962 (3)	0.16836 (19)	0.1020 (11)	
H11A	0.7526	0.3949	0.1475	0.122*	
H11B	0.5733	0.3643	0.1292	0.122*	
C12	0.6101 (3)	0.5072 (2)	0.18016 (13)	0.0691 (6)	
H12	0.7015	0.5514	0.1772	0.083*	
C13	0.5575 (2)	0.52688 (17)	0.26113 (12)	0.0546 (5)	
H13	0.4833	0.5851	0.2518	0.066*	
C14	0.8453 (3)	0.55368 (18)	0.33173 (15)	0.0629 (6)	
H14	0.8818	0.5258	0.2883	0.075*	
C15	0.9531 (3)	0.5882 (2)	0.39677 (19)	0.0804 (8)	
H15	1.0609	0.5831	0.3967	0.097*	
C16	0.9023 (4)	0.6296 (3)	0.46064 (19)	0.0902 (9)	
H16	0.9751	0.6520	0.5044	0.108*	
C17	0.7427 (4)	0.6381 (3)	0.46023 (18)	0.0871 (8)	
H17	0.7072	0.6672	0.5035	0.105*	
C18	0.6351 (3)	0.6036 (2)	0.39563 (15)	0.0709 (7)	
H18	0.5274	0.6097	0.3960	0.085*	
C19	0.6841 (2)	0.55994 (15)	0.33034 (13)	0.0525 (5)	
C20	0.4780 (6)	0.5352 (5)	0.1122 (2)	0.148 (2)	
H20A	0.5080	0.5091	0.0639	0.178*	
H20B	0.3853	0.4964	0.1198	0.178*	
C21	0.4326 (9)	0.6332 (5)	0.0984 (3)	0.208 (4)	
H21A	0.3476	0.6360	0.0534	0.312*	
H21B	0.5202	0.6734	0.0874	0.312*	
H21C	0.3974	0.6608	0.1442	0.312*	
N1	0.4638 (2)	0.43814 (14)	0.28174 (10)	0.0537 (4)	
O1	0.2633 (2)	0.54571 (16)	0.29856 (14)	0.0899 (6)	
Cl1	0.10294 (11)	0.32963 (9)	0.21188 (5)	0.1187 (4)	

### Atomic displacement parameters (Å^2^)

**Table:**

	*U*^11^	*U*^22^	*U*^33^	*U*^12^	*U*^13^	*U*^23^
C1	0.0550 (12)	0.0729 (15)	0.0633 (13)	0.0061 (11)	0.0111 (10)	0.0058 (11)
C2	0.0547 (13)	0.0925 (19)	0.0743 (15)	−0.0028 (12)	0.0100 (11)	0.0089 (14)
C3	0.0616 (12)	0.0538 (12)	0.0591 (12)	−0.0044 (10)	0.0109 (10)	−0.0111 (10)
C4	0.0512 (11)	0.0512 (12)	0.0680 (13)	0.0020 (9)	0.0137 (9)	−0.0013 (10)
C5	0.0824 (17)	0.0577 (14)	0.0891 (19)	−0.0074 (12)	0.0251 (14)	−0.0069 (13)
C6	0.118 (3)	0.0581 (16)	0.117 (3)	−0.0017 (16)	0.043 (2)	0.0175 (17)
C7	0.094 (2)	0.084 (2)	0.094 (2)	0.0109 (16)	0.0215 (17)	0.0320 (17)
C8	0.0758 (16)	0.089 (2)	0.0697 (16)	0.0028 (14)	0.0056 (12)	0.0117 (14)
C9	0.0703 (14)	0.0587 (13)	0.0704 (15)	−0.0040 (11)	0.0055 (11)	0.0014 (11)
C10	0.0902 (18)	0.0597 (14)	0.0897 (18)	0.0065 (12)	0.0419 (15)	−0.0110 (13)
C11	0.147 (3)	0.095 (2)	0.0786 (19)	−0.004 (2)	0.060 (2)	−0.0140 (17)
C12	0.0755 (15)	0.0844 (17)	0.0496 (12)	0.0084 (13)	0.0170 (10)	0.0094 (11)
C13	0.0560 (11)	0.0566 (12)	0.0519 (11)	0.0050 (9)	0.0116 (9)	0.0064 (9)
C14	0.0605 (13)	0.0547 (13)	0.0742 (15)	0.0025 (10)	0.0145 (11)	−0.0003 (11)
C15	0.0621 (14)	0.0729 (17)	0.101 (2)	0.0006 (12)	0.0007 (13)	−0.0113 (15)
C16	0.0839 (19)	0.088 (2)	0.089 (2)	−0.0052 (16)	−0.0108 (15)	−0.0205 (16)
C17	0.094 (2)	0.093 (2)	0.0735 (17)	−0.0028 (16)	0.0130 (14)	−0.0247 (15)
C18	0.0667 (14)	0.0771 (16)	0.0700 (15)	−0.0016 (12)	0.0158 (11)	−0.0124 (13)
C19	0.0591 (12)	0.0421 (10)	0.0565 (11)	0.0014 (8)	0.0112 (9)	0.0053 (9)
C20	0.167 (4)	0.218 (6)	0.0575 (18)	0.076 (4)	0.015 (2)	0.019 (2)
C21	0.292 (8)	0.236 (7)	0.103 (3)	0.176 (7)	0.053 (4)	0.062 (4)
N1	0.0512 (9)	0.0571 (10)	0.0523 (9)	0.0013 (8)	0.0082 (7)	0.0004 (8)
O1	0.0676 (11)	0.0842 (14)	0.1236 (17)	0.0206 (10)	0.0325 (11)	0.0125 (12)
Cl1	0.1024 (6)	0.1474 (9)	0.0951 (6)	−0.0399 (6)	−0.0115 (5)	−0.0055 (5)

### Geometric parameters (Å, º)

**Table:**

C1—O1	1.223 (3)	C11—H11A	0.9700
C1—N1	1.358 (3)	C11—H11B	0.9700
C1—C2	1.507 (4)	C12—C20	1.514 (4)
C2—Cl1	1.760 (3)	C12—C13	1.554 (3)
C2—H2A	0.9700	C12—H12	0.9800
C2—H2B	0.9700	C13—N1	1.480 (3)
C3—N1	1.488 (3)	C13—C19	1.516 (3)
C3—C4	1.512 (3)	C13—H13	0.9800
C3—C10	1.528 (3)	C14—C19	1.384 (3)
C3—H3	0.9800	C14—C15	1.386 (4)
C4—C9	1.385 (3)	C14—H14	0.9300
C4—C5	1.387 (3)	C15—C16	1.358 (4)
C5—C6	1.376 (4)	C15—H15	0.9300
C5—H5	0.9300	C16—C17	1.376 (4)
C6—C7	1.378 (5)	C16—H16	0.9300
C6—H6	0.9300	C17—C18	1.380 (4)
C7—C8	1.366 (4)	C17—H17	0.9300
C7—H7	0.9300	C18—C19	1.384 (3)
C8—C9	1.383 (4)	C18—H18	0.9300
C8—H8	0.9300	C20—C21	1.332 (7)
C9—H9	0.9300	C20—H20A	0.9700
C10—C11	1.507 (4)	C20—H20B	0.9700
C10—H10A	0.9700	C21—H21A	0.9600
C10—H10B	0.9700	C21—H21B	0.9600
C11—C12	1.508 (4)	C21—H21C	0.9600
			
O1—C1—N1	123.3 (2)	C11—C12—C20	107.4 (3)
O1—C1—C2	117.9 (2)	C11—C12—C13	113.4 (2)
N1—C1—C2	118.8 (2)	C20—C12—C13	110.3 (2)
C1—C2—Cl1	109.59 (18)	C11—C12—H12	108.6
C1—C2—H2A	109.8	C20—C12—H12	108.6
Cl1—C2—H2A	109.8	C13—C12—H12	108.6
C1—C2—H2B	109.8	N1—C13—C19	112.11 (16)
Cl1—C2—H2B	109.8	N1—C13—C12	110.22 (19)
H2A—C2—H2B	108.2	C19—C13—C12	117.27 (19)
N1—C3—C4	114.88 (17)	N1—C13—H13	105.4
N1—C3—C10	109.32 (18)	C19—C13—H13	105.4
C4—C3—C10	109.7 (2)	C12—C13—H13	105.4
N1—C3—H3	107.5	C19—C14—C15	121.1 (2)
C4—C3—H3	107.5	C19—C14—H14	119.5
C10—C3—H3	107.5	C15—C14—H14	119.5
C9—C4—C5	118.4 (2)	C16—C15—C14	120.5 (3)
C9—C4—C3	122.6 (2)	C16—C15—H15	119.8
C5—C4—C3	118.9 (2)	C14—C15—H15	119.8
C6—C5—C4	120.6 (3)	C15—C16—C17	119.6 (3)
C6—C5—H5	119.7	C15—C16—H16	120.2
C4—C5—H5	119.7	C17—C16—H16	120.2
C5—C6—C7	120.4 (3)	C16—C17—C18	120.0 (3)
C5—C6—H6	119.8	C16—C17—H17	120.0
C7—C6—H6	119.8	C18—C17—H17	120.0
C6—C7—C8	119.7 (3)	C17—C18—C19	121.3 (2)
C6—C7—H7	120.2	C17—C18—H18	119.3
C8—C7—H7	120.2	C19—C18—H18	119.3
C9—C8—C7	120.3 (3)	C14—C19—C18	117.5 (2)
C9—C8—H8	119.8	C14—C19—C13	124.8 (2)
C7—C8—H8	119.8	C18—C19—C13	117.66 (19)
C8—C9—C4	120.7 (2)	C21—C20—C12	121.1 (5)
C8—C9—H9	119.7	C21—C20—H20A	107.0
C4—C9—H9	119.7	C12—C20—H20A	107.0
C11—C10—C3	110.8 (3)	C21—C20—H20B	107.0
C11—C10—H10A	109.5	C12—C20—H20B	107.0
C3—C10—H10A	109.5	H20A—C20—H20B	106.8
C11—C10—H10B	109.5	C20—C21—H21A	109.5
C3—C10—H10B	109.5	C20—C21—H21B	109.5
H10A—C10—H10B	108.1	H21A—C21—H21B	109.5
C12—C11—C10	113.2 (2)	C20—C21—H21C	109.5
C12—C11—H11A	108.9	H21A—C21—H21C	109.5
C10—C11—H11A	108.9	H21B—C21—H21C	109.5
C12—C11—H11B	108.9	C1—N1—C13	117.42 (18)
C10—C11—H11B	108.9	C1—N1—C3	122.16 (18)
H11A—C11—H11B	107.8	C13—N1—C3	119.97 (16)
			
O1—C1—C2—Cl1	91.4 (3)	C15—C16—C17—C18	0.8 (5)
N1—C1—C2—Cl1	−88.9 (2)	C16—C17—C18—C19	0.0 (5)
N1—C3—C4—C9	−43.6 (3)	C15—C14—C19—C18	1.1 (3)
C10—C3—C4—C9	80.1 (3)	C15—C14—C19—C13	178.3 (2)
N1—C3—C4—C5	140.4 (2)	C17—C18—C19—C14	−1.0 (4)
C10—C3—C4—C5	−96.0 (2)	C17—C18—C19—C13	−178.3 (3)
C9—C4—C5—C6	−1.0 (4)	N1—C13—C19—C14	115.1 (2)
C3—C4—C5—C6	175.2 (2)	C12—C13—C19—C14	−13.9 (3)
C4—C5—C6—C7	1.1 (5)	N1—C13—C19—C18	−67.7 (3)
C5—C6—C7—C8	−0.2 (5)	C12—C13—C19—C18	163.3 (2)
C6—C7—C8—C9	−0.7 (5)	C11—C12—C20—C21	−167.8 (5)
C7—C8—C9—C4	0.7 (4)	C13—C12—C20—C21	68.1 (6)
C5—C4—C9—C8	0.1 (4)	O1—C1—N1—C13	−8.1 (3)
C3—C4—C9—C8	−175.9 (2)	C2—C1—N1—C13	172.09 (19)
N1—C3—C10—C11	−49.6 (3)	O1—C1—N1—C3	179.6 (2)
C4—C3—C10—C11	−176.4 (2)	C2—C1—N1—C3	−0.2 (3)
C3—C10—C11—C12	60.0 (4)	C19—C13—N1—C1	104.6 (2)
C10—C11—C12—C20	−135.7 (3)	C12—C13—N1—C1	−122.8 (2)
C10—C11—C12—C13	−13.6 (4)	C19—C13—N1—C3	−83.0 (2)
C11—C12—C13—N1	−38.1 (3)	C12—C13—N1—C3	49.6 (2)
C20—C12—C13—N1	82.3 (3)	C4—C3—N1—C1	−69.3 (3)
C11—C12—C13—C19	91.8 (3)	C10—C3—N1—C1	166.8 (2)
C20—C12—C13—C19	−147.8 (3)	C4—C3—N1—C13	118.6 (2)
C19—C14—C15—C16	−0.3 (4)	C10—C3—N1—C13	−5.2 (3)
C14—C15—C16—C17	−0.7 (5)		
