# Supplementary figures and images for: Crystal structure of 2-chloro-1-(3-ethyl-2,6-di­phenyl­piperidin-1-yl)ethanone
Source: Acta Crystallogr E Crystallogr Commun. 2015 Jan 21;71(Pt 2):o122. doi: 10.1107/S2056989015000444 (PMC4384592; doi:10.1107/S2056989015000444)

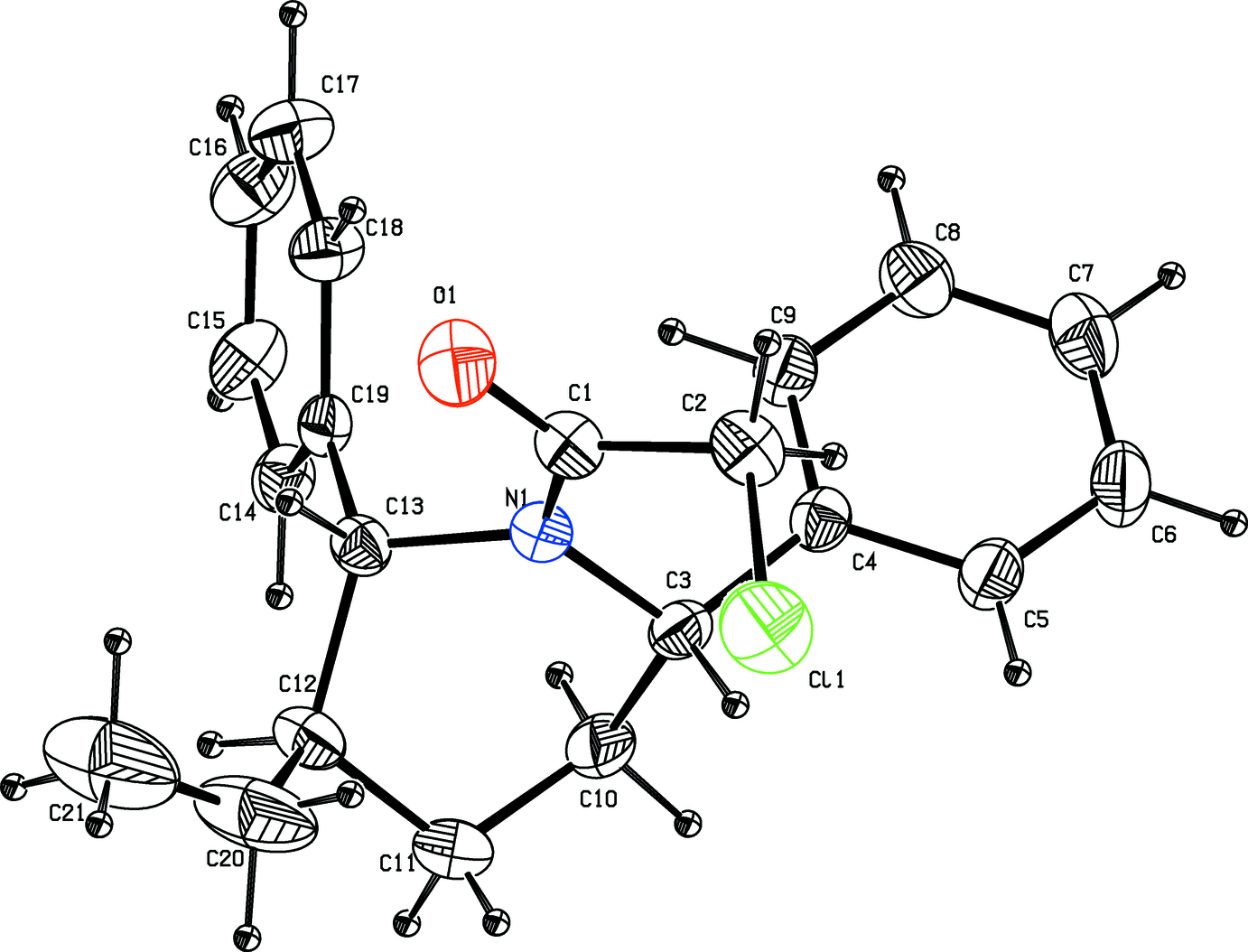

Supplement: Supplementary file 4 [file e-71-0o122-fig1.tif]

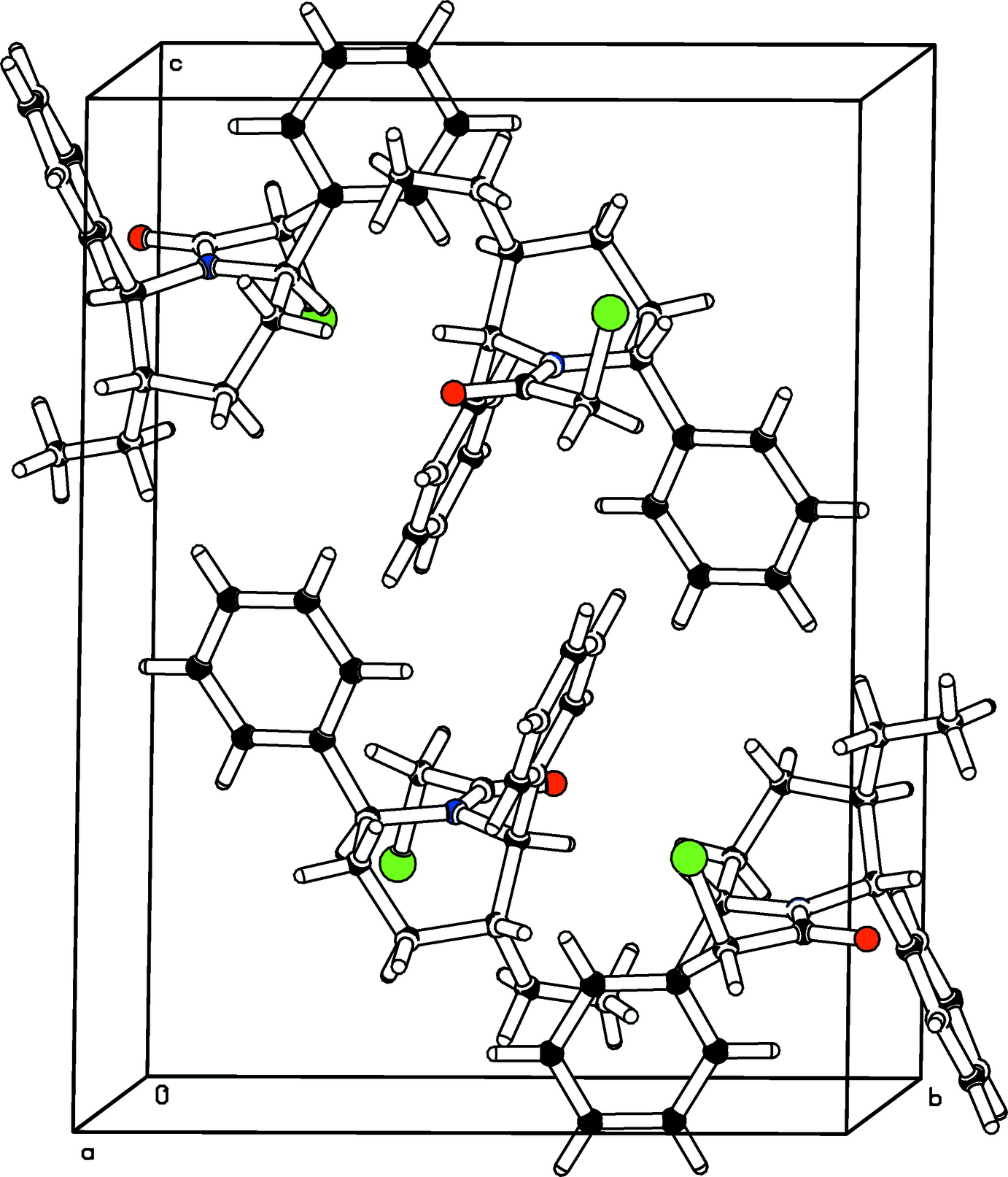

Supplement: Supplementary file 5 [file e-71-0o122-fig2.tif]
